# College and Hospital Notes

**Published:** 1897-02

**Authors:** 


### College and Hospital Notes.

The Bellevue Hospital Medical College building at New York, situated upon the hospital grounds, was very nearly destroyed by fire Wednesday morning, January 20, 1897. Frank Murphy, the gatekeeper of the hospital, discovered the flame at five o'clock and rang an alarm, after which he hastened to the hospital office and gave notice of the fire. No person was in the college when the flames were discovered, but a number of hospital attendants and the men employed at the morgue aided the firemen and policemen in saving valuable books and documents. The dispensary, in the basement of the building, was saved from serious damage, but the upper part of the structure was burned out completely. The valuable records, protected in safes on the first and second floors, were preserved. The lecture room and auditorium in the third story were completely wrecked, and the dissecting room was burned out, including much material in use by the anatomical classes. After the fire was extinguished many books from the library were removed to the hospital office for preservation.

The building was erected in 1865, and the damage by this fire is estimated at about $20,000, besides the loss of instruments, specimens in the museum and furniture, which will probably increase the total loss to $30,000, which is only partly covered by insurance.

The fire presumptively arose from a defective electric light wire. It is stated that the building will be repaired as speedily as possible. Meanwhile the lectures are being conducted at the Carnegie laboratory. Dr. Austin Flint, secretary of the faculty, has announced that the fire would not interfere with the work of the college.

Although the glare of the flames of the burning building was visible in several of the hospital wards, there was no alarm among the 755 patients. All the physicians, orderlies and attendants of the hospital were soon on duty in the wards, and every precaution was taken to prevent a panic. Even in the insane pavilion, which was nearest to the burning building, the patients remained on their cots and were not frightened. Much credit is due to the officers in charge for discreet management on this trying occasion.

---

The board of directors of the Pennsylvania society for the prevention of tuberculosis has secured a suitable site for the proposed new state sanitarium for the cure of consumption. The site is situated near White Haven, in Luzerne county, within close proximity to Glen Summit, and covers an area of nearly 600 acres.

A committee consisting of Dr. H. M. Anders, Dr. Samuel G. Dixon, Miss Redfield, James L. Stanton and Dr. S. Solis-Cohen has been appointed to raise the sum of $15,000 for the erection of the necessary building.

